# Intervention in Health Misinformation Using Large Language Models for Automated Detection, Thematic Analysis, and Inoculation: Case Study on COVID-19

**DOI:** 10.2196/75500

**Published:** 2026-01-08

**Authors:** Samira Malek, Christopher Griffin, Robert D Fraleigh, Robert Lennon, Vishal Monga, Lijiang Shen

**Affiliations:** 1 Department of Computer Science and Engineering, Pennsylvania State University, University Park, PA, United States; 2 Applied Research Laboratory, Pennsylvania State University, University Park, PA, United States; 3 Department of Mathematics, Pennsylvania State University, University Park, PA, United States; 4 PrimeCare Medical, Harrisburg, PA, United States; 5 Department of Electrical Engineering, Pennsylvania State University, University Park, PA, United States; 6 Department of Communication Arts and Sciences, Pennsylvania State University, 211 Sparks Building, University Park, PA, 16802, United States, 1 (814) 865-1736

**Keywords:** large language models, topic modeling, COVID-19, misinformation, prompt engineering, machine learning

## Abstract

**Background:**

The rapid growth of social media as an information channel has enabled the swift spread of inaccurate or false health information, significantly impacting public health. This widespread dissemination of misinformation has caused confusion, eroded trust in health authorities, led to noncompliance with health guidelines, and encouraged risky health behaviors. Understanding the dynamics of misinformation on social media is essential for devising effective public health communication strategies.

**Objective:**

This study aims to present a comprehensive and automated approach that leverages large language models (LLMs) and machine learning techniques to detect misinformation on social media, uncover the underlying causes and themes, and generate refutation arguments, facilitating control of its spread and promoting public health outcomes by inoculating people against health misinformation.

**Methods:**

We use 2 datasets to train 3 LLMs, namely, BERT, T5, and GPT-2, to classify documents into 2 categories: misinformation and nonmisinformation. In addition, we use a separate dataset to identify misinformation topics. To analyze these topics, we applied 3 topic modeling algorithms—Latent Dirichlet Allocation, Top2Vec, and BERTopic—and selected the optimal model based on performance evaluated across 3 metrics. Using a prompting approach, we extract sentence-level representations for the topics to uncover their underlying themes. Finally, we design a prompt text capable of identifying misinformation themes effectively.

**Results:**

The trained BERT model demonstrated exceptional performance, achieving 98% accuracy in classifying misinformation and nonmisinformation, with a 44% reduction in false-positive rates for artificial intelligence–generated misinformation. Among the 3 topic modeling approaches used, BERTopic outperformed the others, achieving the highest metrics with a Coherence Value of 0.41, Normalized Pointwise Mutual Information of −0.086, and Inverse Rank-Biased Overlap of 0.99. To address the issue of unclassified documents, we developed an algorithm to assign each document to its closest topic. In addition, we proposed a novel method using prompt engineering to generate sentence-level representations for each topic, achieving a 99.6% approval rate as “appropriate” or “somewhat appropriate” by 3 independent raters. We further designed a prompt text to identify themes of misinformation topics and developed another prompt capable of detecting misinformation themes with 82% accuracy.

**Conclusions:**

This study presents a comprehensive and automated approach to addressing health misinformation on social media using advanced machine learning and natural language processing techniques. By leveraging LLMs and prompt engineering, the system effectively detects misinformation, identifies underlying themes, and provides explanatory responses to combat its spread. The proposed method was tested on an English language COVID-19–related dataset and has not been evaluated on real-world online social media data; the experiments were conducted offline.

## Introduction

Misinformation and inaccurate beliefs and knowledge about health can substantially undermine well-being by fueling confusion, eroding trust in reliable medical advice, and prompting risky behaviors such as rejecting vaccines, turning to scientifically unproven home remedies, or neglecting protective measures amid clear dangers [1-8]. These inaccuracies often circulate rapidly via social media, exploiting emotional narratives that overshadow fact-based content and leading individuals to question the legitimacy of evidence-based interventions [5,8-11]. Repeated exposure to misinformation reduces health literacy and can reinforce people’s belief in falsehoods, making them more likely to view credible health authorities with skepticism [12-14]. As a result, misinformation weakens the success of prevention and treatment strategies, paving the way for heightened disease transmission, avoidable complications, and deteriorating outcomes at both individual and community levels [15-18].

An illustration comes from the COVID-19 pandemic, which saw an unprecedented surge of misinformation and conspiracy theories—labeled an “infodemic” by the World Health Organization (WHO) [1,12]. False remedies, unverified claims on the origins of the virus, and politicized narratives about preventive measures severely hampered containment efforts [19-21]. While proven strategies such as mask wearing, vaccination, and physical distancing were promoted by scientific authorities, social media rumors cast doubt on vaccine safety and the reality of the virus itself, discouraging people from getting vaccinated or seeking appropriate medical care [2,22-24]. This breakdown in adherence prolonged outbreaks, overloaded health infrastructures, and ultimately jeopardized global health and economic stability [25].

A parallel can be drawn from discussions around the human papillomavirus (HPV) vaccine, which has proven crucial in preventing various HPV-related cancers, including cervical cancer that claims thousands of lives each year [26-29]. Widespread misinformation about adverse effects and conspiracies regarding its necessity led to a significant portion of unvaccinated adolescents, heightening the likelihood of HPV infection and future malignancies [30]. This trend not only increased the burden on public health systems but also underscored the power of misinformation to undermine trust in legitimate medical counsel.

In recent years, social media has become a central and highly accessible source of information for millions of users worldwide [31]. However, its ability to rapidly disseminate content—including unfounded claims—creates fertile ground for large-scale propagation of misinformation. Given the sheer volume of posts, manual monitoring and analysis of such content are impractical [31,32] Consequently, developing and using automated, data-driven methods to understand and manage the dynamics of digital misinformation are essential for preserving accurate information and safeguarding public trust.

In this study, we propose an automated system designed to identify whether a given text contains misinformation. If misinformation is detected, the system analyzes the theme of the misinformation and provides a refutation argument (inoculation) to help prevent its spread on social media and enhance public health awareness. To achieve this, we leverage a large language model (LLM) to detect misinformation effectively. Furthermore, we demonstrate that enriching datasets significantly improves the detection of misinformation generated by both humans and AI. Recent advances in LLMs, such as ChatGPT, have enabled the generation of increasingly sophisticated misinformation, which poses challenges for traditional machine learning (ML) methods in distinguishing AI-generated misinformation [33,34]. While prior research has highlighted the effectiveness of deep learning methods in classifying health-related misinformation, these efforts have predominantly focused on content generated by humans [35,36]. Moreover, our proposed process generates sentence-level descriptions of misinformation topics, eliminating the need for manual interpretation. However, prior approaches relied on ML-based methods that produced word-level topic representations, which required manual interpretation to form coherent sentence-level topics—introducing potential human errors and subjective biases [1,22]. Similar challenges arise in other ML-based applications, such as optimizing models in industries where manual calibration of parameters can lead to inefficiencies and errors. For example, recent research has demonstrated that data-driven models can enhance predictive accuracy and automate decision-making, reducing human intervention in systems that rely on complex data streams [37-40]. Inspired by these advances, our process generates sentence-level descriptions of misinformation topics, eliminating the need for manual interpretation. In addition, we introduce an algorithm to assign documents to the most relevant topics. This addresses the limitation of many ML-based topic modeling algorithms, which often leave some documents unclassified. Our process also identifies overarching themes of misinformation topics automatically, providing a high-level understanding of the underlying reasons for misinformation categorization. Although the COVID-19 pandemic serves as our illustrative case due to its scale and data availability, the underlying challenges we address—rapid online spread, emotionally charged narratives, and declining trust—are common across other health contexts (eg, HPV vaccines) and beyond. Our approach does not rely on COVID-specific lexicons or handcrafted rules. Instead, the proposed Misinformation Detection and Inoculation Process (MDIP) is domain-agnostic. It ingests free English text, induces topics using standard models, transforms word lists into sentence-level descriptors through targeted LLM prompting, organizes them into hierarchical themes (guided by coherence, diversity metrics, and generic embeddings), and maps themes to refutation templates. Each stage has the potential to be applied to other health domains and misinformation settings, provided that the AI is properly trained with the topic- or domain-specific data.

Many previous studies have focused on individual aspects of the misinformation problem, such as detection or topic analysis [1,35,41], but have not integrated these steps into a unified framework for intervention. Our MDIP and Misinformation Detection and Inoculation System (MDIS) frameworks unify misinformation detection, topic modeling, thematic refutation, and public health intervention into a single, automated workflow. This end-to-end approach enables health teams to move beyond merely identifying misinformation to actively and effectively countering it.

In the study by He et al [42], a method was proposed to generate per-claim counterresponses. While generating responses tailored to each specific piece of misinformation can be more informative and persuasive, such approaches require paired datasets of misinformation posts and response arguments for model training—datasets that are difficult to construct. Moreover, misinformation often mutates through paraphrasing and subtle edits; claim-specific pipelines are fragile in the face of such variation [43,44]. By contrast, our theme-level refutation approach is robust to these surface changes. More recently, LLM-based topic modeling approaches, such as TopicGPT [45] and other methods [46,47], have leveraged the capabilities of powerful pretrained models such as ChatGPT and LLaMA. While these methods benefit from the models’ deep understanding of language, they often require passing entire documents through parameter-rich models, which leads to increased latency and computational costs compared with traditional pipelines. Furthermore, they typically yield only single-level sets of word topics. In contrast, our hybrid framework balances efficiency and expressiveness: we first use a traditional topic inducer to efficiently uncover the underlying structure and then apply LLM prompting (via ChatGPT) where it provides the added value. Specifically, the LLM is used to transform word lists into sentence-level topic labels and to organize topics into hierarchical themes. This targeted use of LLMs preserves computational efficiency while producing richer, hierarchical, and deployment-ready representations that are well suited for downstream tasks such as detection, monitoring, and refutation.

## Methods

### Study Design

In this study, we propose the MDIP, a comprehensive framework designed to analyze the dynamics of misinformation automatically and develop an MDIS. The MDIS end-to-end pipeline (1) flags misinformation, (2) explains what it is about via topics and higher-level themes, and (3) returns a concise, theme-matched refutation. The components are modular and feed one another in a simple data flow. No step in MDIP and MDIS uses disease-specific features; inputs are raw text and model hyperparameters chosen by intrinsic criteria (topic coherence and diversity). This makes the pipeline directly applicable to other misinformation corpora after swapping in the relevant documents. The MDIP framework is structured into four interconnected sections, each addressing a critical aspect of misinformation management:

1. *Detect misinformation*: Collect a labeled dataset and then train LLMs to classify text documents as either misinformation or nonmisinformation, providing a foundation for identifying false narratives.
2. *Misinformation topics*: Here, a topic modeling algorithm is applied to uncover the key topics within misinformation datasets. This step helps categorize misinformation into specific subject areas, enabling a better understanding of its thematic structure.
3. *Topic descriptions*: This section uses prompt engineering and the results of the previous section to enhance interpretability by generating sentence-level representations for each topic, moving beyond traditional word-level outputs. These descriptive summaries provide meaningful context for understanding the essence of each topic.
4. *Provide refutation*: In the final step, topic descriptions and extracted themes are used to design a specific prompt that identifies the underlying themes of misinformation. The system then generates clear and contextually relevant refutation arguments tailored to the detected misinformation themes. These arguments are designed to counter false narratives, improve public understanding, and mitigate the spread of misinformation. The refutations are a key component in the psychological inoculation-based misinformation mitigation intervention. As a metaphor to medical vaccination, the typical inoculation strategy consists of an attack message (ie, as a small weakened or deactivated dose of the virus) and refutation or counterarguments against the attack message (ie, as the immune system’s reaction to the vaccine when it is injected or otherwise enters the human body) [48].

**Figure 1.**
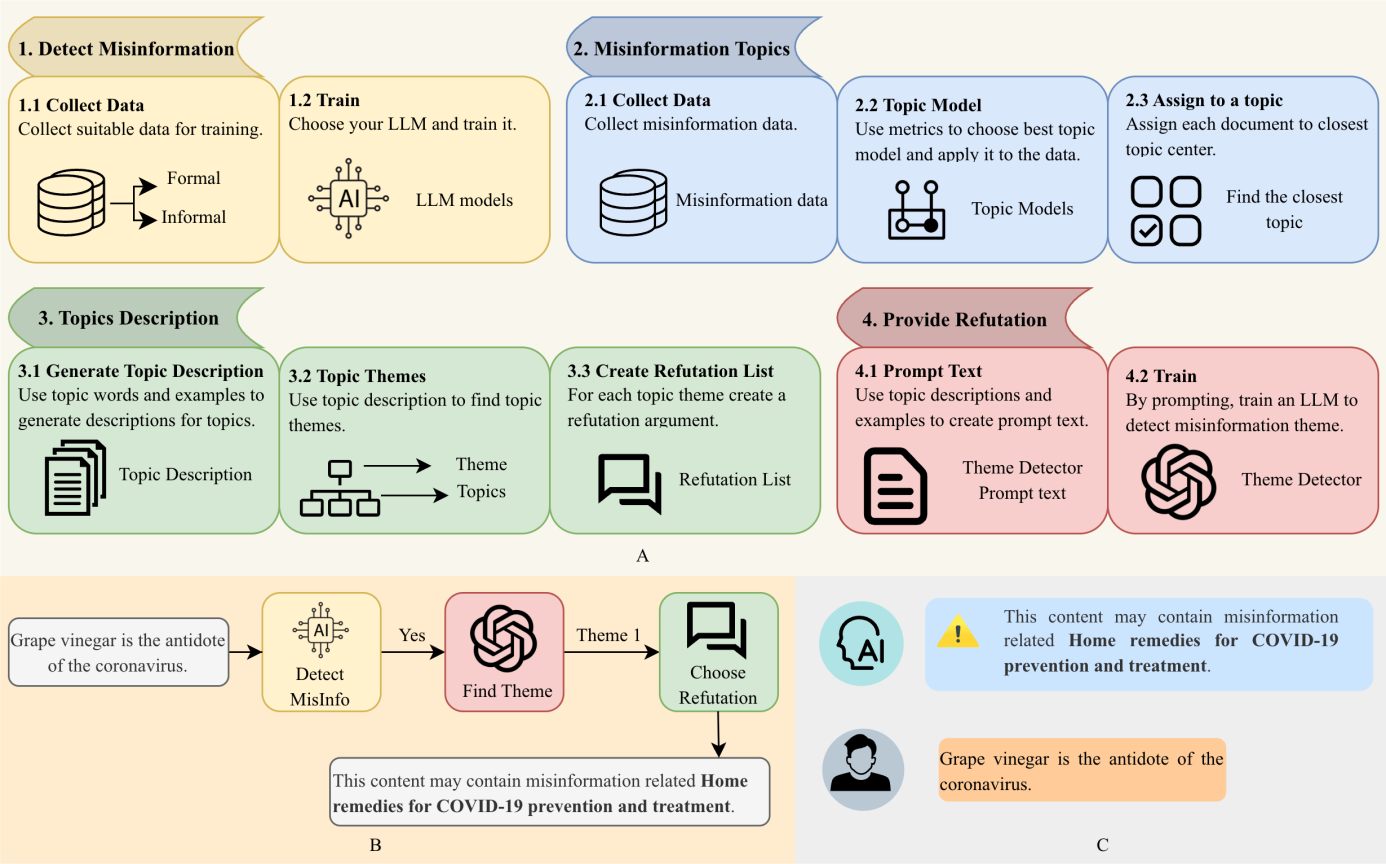
(**A**) Overview of the Misinformation Detection and Inoculation Process (MDIP) integrates four main stages: (1) misinformation detection through supervised classification, (2) topic modeling of misinformation texts and assignment of outliers, (3) generation of interpretable topic descriptions and aggregation into higher-level themes, and (4) theme detection and provision of theme-linked refutations via prompt engineering for detected misinformation. (**B**) Misinformation Detection and Inoculation System (MDIS) workflow: For any new text, the system outputs (1) the misinformation decision, (2) its most likely theme, and (3) a theme-matched refutation. (**C**) Example: Illustration of how the system delivers the final user-facing output, providing a warning about misinformation and the matched refutation text. LLM: large language model.

By integrating these components, MDIP enables the development of MDIS, an intelligent and automated system capable of detecting misinformation, identifying its themes, and delivering refutations to combat its impact on public health. The overall architecture of the proposed framework is illustrated in Figure 1. Figure 1A outlines the 4 stages of the MDIP: supervised detection of misinformation, topic modeling, generation of interpretable topic descriptions, and theme-based refutation. Figure 1B presents the end-to-end workflow of the MDIS, which processes new text inputs to produce a misinformation classification, assign the text to a thematic category, and generate a matched refutation. Finally, Figure 1C provides an example of the user-facing output, demonstrating how the system delivers both a misinformation warning and a concise, contextually relevant refutation.

### Detect Misinformation

Misinformation detection in text documents has become a critical area of research due to the growing prevalence of misleading or false information online. To address this challenge, we use classifiers based on LLMs. These LLMs are trained to categorize text into 2 classes: misinformation and nonmisinformation, as shown in the first part of Figure 1A.

Our classifier was trained using 2 complementary datasets, each providing diverse linguistic characteristics to enhance performance in detecting misinformation. The first dataset, the AAAI 2021 Competition Dataset [49], consists of misinformation sourced from social media platforms such as Facebook and X (formerly known as Twitter). This dataset reflects the informal, conversational style of social media, characterized by casual tone, nonstandard grammar, and the use of slang. The second dataset, COVID_19FNIR [50], includes misinformation presented in formal, structured language, offering a stark contrast to the informal nature of the first dataset. By incorporating these 2 datasets, we trained 3 different LLMs to detect misinformation effectively across a wide spectrum of communication styles. The blend of informal and formal language enabled the model to better generalize, achieving improved accuracy and robustness in identifying misinformation, whether generated by humans or artificial intelligence (AI).

Traditionally, researchers collect human-written data from social media platforms such as Twitter and Facebook, label them as misinformation or nonmisinformation, and then train a deep neural network to classify such documents [35,36,51,52]. However, recent studies have demonstrated that deep neural networks trained exclusively on human-written datasets exhibit weaker accuracy in detecting AI-generated misinformation compared with human-written misinformation. This discrepancy arises because AI-generated misinformation often adopts formal language styles similar to accurate information shared by credible sources such as the WHO and the Centers for Disease Control and Prevention (CDC) on official social media accounts [33,34].

In this research, by combining a dataset with formal language and another with informal language (enriching the dataset with different language types and more misinformation), we demonstrate that LLMs achieve reasonable accuracy in detecting AI-generated misinformation. This approach ensures better generalization and robustness, bridging the gap in identifying misinformation across diverse linguistic styles.

### Misinformation Topics

As outlined in the second section of Figure 1A, our approach involves 3 key steps. First, we collect misinformation data. Next, we select and compare topic modeling algorithms based on specific features and metrics to identify the most effective model. Finally, we design an algorithm that assigns topics to new or unclassified documents.

To identify misinformation topics, we used one of the largest datasets of verified COVID-19 claims, the IFCN dataset, which has been extensively used in related research [41,53,54]. We applied 3 topic modeling algorithms—Latent Dirichlet Allocation (LDA) [55], Top2Vec [56,57], and BERTopic [57,58]—to analyze this dataset.

To evaluate and compare the performance of these algorithms, we selected 3 metrics: Coherence Value (CV) [59], Normalized Pointwise Mutual Information (NPMI) [59], and Inverse Rank-Biased Overlap (IRBO) [60]. CV and NPMI measure the coherence of the topics, ensuring that they are logically consistent, being human interpretable, and meaningful. IRBO, on the other hand, evaluates the diversity of the topics generated by the model, which is crucial for ensuring broad coverage of the dataset’s content. Since our focus is on misinformation within health-related social media data, coherence and diversity are particularly important to ensure that topics are both interpretable and representative.

After selecting the best-performing topic model, we developed an algorithm to address the issue of unclassified documents. This algorithm assigns topics to new or previously unassigned documents, ensuring comprehensive topic coverage and improved usability of the model for real-world applications.

### Topic Description

Topic modeling algorithms typically produce word-level representations for each topic. While these representations provide insight into the most relevant words associated with a topic, they often lack the semantic depth necessary to precisely identify the specific topic within a document. This limitation arises because word-level outputs fail to capture the context and relationships between words that define the overarching theme of a topic [51].

Recent advancements in LLMs have demonstrated their ability to generate high-quality, contextually relevant outputs with minimal or zero additional training by designing carefully crafted inputs—referred to as prompt engineering [61]. Leveraging this capability, we address the limitations of word-level representations by using prompt engineering techniques to generate sentence-level representations for each topic. These sentence-level representations capture the context and essence of the topic, enabling a more accurate and interpretable understanding of the document content.

Subsequently, these sentence-level representations are used to identify and articulate the overarching themes of the topics, also at the sentence level. This approach provides a more comprehensive view of the thematic structure within the document corpus. Finally, recognizing that all documents within the dataset share a common underlying reason for being classified as misinformation, we develop a tailored response list for each topic theme. The third section of Figure 1A illustrates these 3 steps.

### Provide Refutation

In the final step of our proposed method, as illustrated in the final part of Figure 1A, we identify the overarching theme of misinformation and provide a corresponding response from a preconstructed response list. This response list is developed in the preceding step based on the identified themes.

To determine the themes of misinformation, we use prompt engineering techniques. By designing carefully crafted and contextually appropriate prompt text, we effectively extract the underlying themes associated with misinformation. This approach allows us to translate complex word-level or sentence-level representations into meaningful thematic insights.

By identifying misinformation themes and providing precise, theme-based responses, our method aims to enhance public health knowledge and reduce the spread of misinformation. This proactive approach not only mitigates the risks associated with false or misleading information but also fosters a more informed and resilient society.

### Proposed System

Following the completion of four foundational steps—(1) detecting misinformation, (2) identifying misinformation topics, (3) describing topics, and (4) providing refutations—we develop our comprehensive MDIS, which consists of three key components.

1. *Detection of misinformation*: The system begins by determining whether a given document is misinformation.
2. *Identification of misinformation themes*: If the document is classified as misinformation, the system analyzes its content to identify the underlying misinformation themes. This process involves extracting thematic representations that provide a clearer understanding of the document’s misleading aspects.
3. *Providing refutations*: Finally, the system generates a detailed refutation argument for the identified misinformation themes. These arguments are derived from a predesigned response list tailored to address specific misinformation themes effectively.

All 3 components of the system are demonstrated with a practical example, as illustrated in Figure 1B and C. This example highlights how the system operates cohesively to detect misinformation, uncover its thematic structure, and deliver accurate refutations, ultimately contributing to a more informed and resilient public.

### Ethical Considerations

This study did not involve human participants, human tissue, or the collection of identifiable private information by the authors. All analyses were conducted on previously collected, publicly available, and deidentified datasets, obtained solely for research purposes. Specifically, the data sources include: (1) the AAAI 2021 COVID-19 Fake News Detection Competition dataset, originally released as part of the AAAI Conference on Artificial Intelligence shared task, in which all social media content was anonymized and distributed for noncommercial research use only [49]; (2) the COVID-19 FNIR (Fake News and Information Reliability) dataset, introduced by prior studies for misinformation detection research and released in deidentified form for academic use [50]; and (3) the International Fact-Checking Network (IFCN) COVID-19 fact-checking corpus, which aggregates publicly available fact-check articles produced by IFCN-certified organizations and contains no personal or sensitive individual-level data [62]. According to the US Department of Health and Human Services Common Rule (45 CFR §46.104(d)), secondary research involving publicly available, deidentified data does not constitute human subjects research and is therefore exempt from Institutional Review Board review [63]. The research complied with all relevant ethical standards and data use policies and poses no risk to individuals or communities. The study’s sole objective is to advance computational methods for understanding and mitigating the spread of health misinformation.

## Results

### Text Classification

Due to the exceptional performance of LLMs across a wide range of AI tasks, we leveraged 3 prominent LLMs to fine-tune them for COVID-19 text classification. These models—BERT (Bidirectional Encoder Representations from Transformers), GPT-2 (Generative Pre-trained Transformer 2), and T5-base (Text-to-Text Transfer Transformer)—are renowned for their ability to understand and process natural language with high accuracy and contextual awareness.

BERT is particularly effective in handling text classification tasks due to its bidirectional context understanding, which allows it to capture nuanced language patterns [64]. GPT-2 excels in text generation and classification by leveraging its autoregressive architecture to predict sequences in a given context [65]. Finally, T5-base under a unified framework that reformulates all NLP tasks as a text-to-text problem, making it versatile and effective across various domains [66].

To conduct this study, we combined the AAAI 2021 competition dataset with the COVID-19 FNIR dataset. The data were split into training, testing, and validation sets with proportions of 67%, 17%, and 16%, respectively.

Accuracy, *F*_1_-score, Recall, and Precision are standard metrics for evaluating classification models. Accuracy measures the proportion of all predictions that are correct, providing an overall performance indicator but sometimes masking class imbalances. Precision quantifies the fraction of predicted positives that are truly positive, reflecting how often the model avoids false alarms. Recall (or sensitivity) measures the fraction of actual positives that the model successfully identifies, highlighting its ability to capture relevant cases [67]. *F*_1_-score is the harmonic mean of precision and recall, balancing the trade-off between the two. In health-related text classification tasks such as misinformation detection, reasonable thresholds are often set required due to the risks of misclassification—for instance, aiming for Accuracy >0.80, *F*_1_-score ≥0.75, Recall ≥0.75, and Precision ≥0.70—to ensure both reliable detection and practical usability in downstream inoculation public health applications. Table 1 shows the evaluation metrics, including Accuracy, *F*_1_-score, Recall, and Precision, for all 3 models on the test dataset. Among these, BERT achieved the highest performance, with an accuracy of *98%* on the test data. This result highlights BERT’s ability to handle complex linguistic structures and its effectiveness in fine-tuning for domain-specific tasks such as COVID-19 text classification.

**Figure 2.**
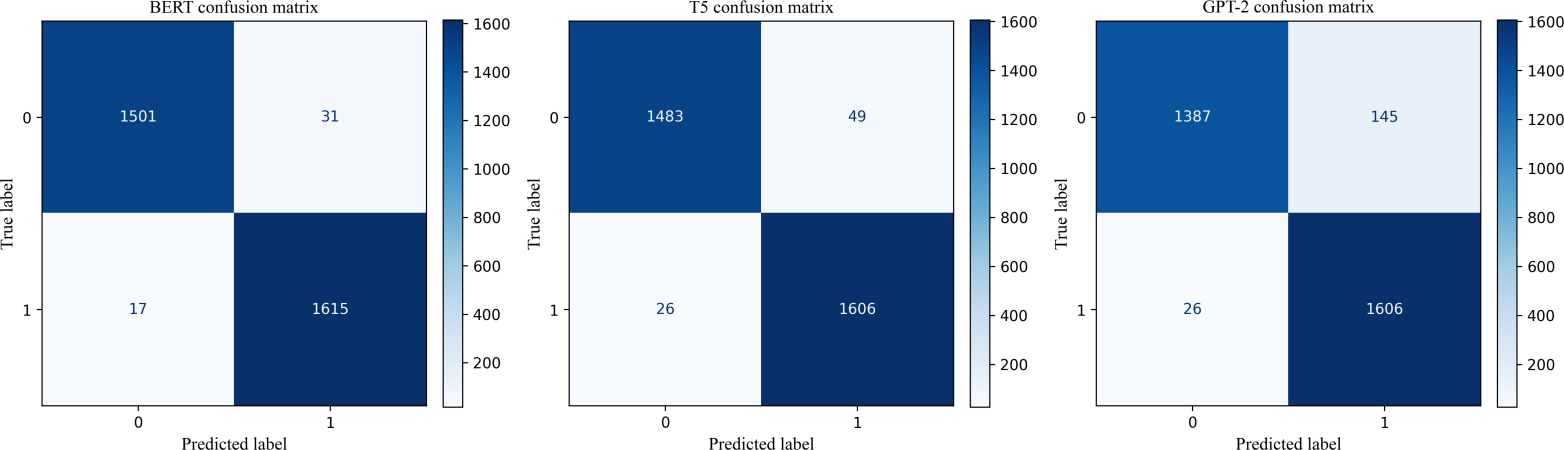
Confusion matrices illustrate the performance of the 3 binary classification models (BERT, GPT-2, and T5-base). GPT-2: Generative Pre-trained Transformer.

The confusion matrices in Figure 2 for BERT, GPT-2, and T5-base further illustrate the performance of these models, providing a detailed breakdown of true positives, true negatives, false positives, and false negatives, which helps in understanding their classification strengths and potential areas for improvement.

**Table 1.** Performance metrics (Accuracy, *F*_1_-score, Recall, and Precision) on the test dataset for 3 models: BERT-base, GPT-2, and T5-base.

Model	Accuracy	*F*_1_-score	Recall	Precision
BERT	0.9848	0.9854	0.9896	0.9812
GPT-2^a^	0.9460	0.9495	0.9841	0.9117
T5-base (Generic Condition)	0.9763	0.9763	0.9763	0.9764

a GPT-2: Generative Pre-trained Transformer.

**Figure 3.**
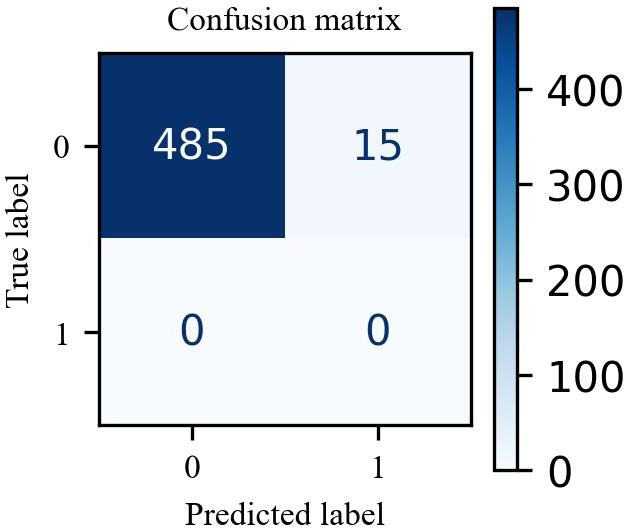
The figure displays the confusion matrix of our fine-tuned BERT model evaluated on the artificial intelligence–generated dataset [3].

To evaluate the accuracy of our model on AI-generated data, we used the dataset provided in the study by Du et al [35]. We tested our fine-tuned BERT model on this dataset, and the results are shown in Table 2. The findings indicate a significant reduction in the number of false positives, decreasing from 27 to 15, representing a 44% improvement. In addition, Figure 3 shows the confusion matrix for our fine-tuned BERT model when applied to the AI-generated misinformation dataset.

**Table 2.** False-positive and true-negative results obtained from testing the fine-tuned BERT model on our combined dataset, compared with the results reported in the study by Zhou et al [33].

Model	FP^a^	TN^b^
Our	15	485
Zhou et al [33]	27	473

a FP: false-positive.

b TN: true-negative.

### Topic Models

We used the IFCN dataset, one of the largest datasets on COVID-19 pandemic, to apply and evaluate topic modeling approaches. Three models were tested: LDA, Top2Vec, and BERTopic. After applying each topic model, the top 10 words associated with each topic were selected, and the 3 metrics—CV, NPMI, and IRBO—were computed to compare the models. Table 3 summarizes the results across these metrics. Among the models, BERTopic achieved the highest scores across all metrics, leading to its selection for further analysis. In practice, the reported metrics indicate that the topics are moderately coherent and interpretable but not perfectly tight. A coherence score of 0.41 means that the top words in each topic tend to appear together often enough for human analysts to assign clear labels, although some mixing of subthemes is expected. The NPMI of −0.086 is close to neutral, which is typical for short, fragmented social media posts, and suggests that while not every word pair strongly co-occurs, the overall topics remain meaningful. Combined with the high IRBO score (0.99), these results imply that the model generates a broad, nonredundant set of topics that cover diverse misinformation themes while remaining practically usable for labeling, interpretation, and downstream health communication tasks. Since in the health-related domain, it is important to cover as many diverse topics as possible, an IRBO value above 0.7 can be considered acceptable, while a CV value equal to or above 0.4 and an NPMI close to 0 or higher are reasonable.

**Table 3.** Performance of 3 topic modeling approaches—LDA, Top2Vec, and BERTopic—evaluated across 3 metrics: Coherence Value, Normalized Pointwise Mutual Information, and Inverse Ranked Based Overlap^a^.

Model	CV­^b^	NPMI^c^	IRBO^d^ ­
LDA	0.39	−0.35	0.96
Top2Vec	0.35	−0.29	0.89
BERTopic	0.41	−0.086	0.99

a For all metrics, higher values indicate better performance.

b CV: Coherence Value.

c NPMI: Normalized Pointwise Mutual Information.

d IRBO: Inverse Ranked Based Overlap.

Many topic modeling approaches, including BERTopic, often encounter limitations when applied to real-world datasets, as they are unable to assign topics to all documents. This can leave a subset of documents unclassified, reducing the overall effectiveness of the model. To address this issue, we have used the algorithm in Textbox 1, a method for ensuring comprehensive topic assignment across the dataset [68].

Textbox 1. **Algorithm: assign a document to the closest topic.**

1. Input:
  - Raw text documents $X=\left\{d_{1},d_{2},...,d_{n}\right\}$,
  - BERTopic model parameters.
2. Topic modeling:
  - Compute topics using the BERTopic model:
    Topics ${\left\{Y_{j}\right\}}_{j=1}^{T}$, = BERTopic(*X,P*)
    where *T* is the number of topics and *Y*_*j*_ contains documents that are assigned to the topic *j*.
3. Sentence embeddings:
  - Transform documents *X* into vector representations using a sentence transformer such as BERT embedding.
4. Dimensionality reduction:
  - Apply Uniform Manifold Approximation and Projection for dimensionality reduction on the vector representations.
5. Cluster centers:
  - For each topic *j*, compute the center of the cluster:

(1) $$t_{j}=\sum_{i\in j}\frac{x_{i}}{n_{j}}$$

where *n*_*j*_ is the number of documents in topic *j*, and *x_i_* is the reduced vector representation of document.

1. Topic assignment for unassigned documents:
  - For every document *d_i_* that is not assigned to a topic by the BERTopic model, or for any new document:
    - Assign the document to the topic *j* that maximizes the cosine similarity between the document vector *x_i_* and the cluster center *t_j_* :

(2) $$Argmax{\;}_{j}\frac{x_{i}\;\cdot \;t_{j}}{\left\|x_{i}\right\|\left\|t_{j}\right\|}$$

This approach not only ensures that every document in the dataset is assigned a topic but also enhances the interpretability and usability of the topic modeling results. By leveraging the semantic structure of the dataset, our algorithm effectively bridges the gap between unassigned documents and existing topic clusters, making it a robust solution for comprehensive topic coverage.

### Topic Description

As described in the “Methods” section, topic modeling algorithms typically produce word-level representations of topics. While useful for identifying key terms associated with a topic, these representations often lack sufficient contextual information, making interpretation challenging. To overcome this limitation, we developed a structured prompt framework (outlined in Textbox 2 and used the advanced capabilities of LLMs, specifically ChatGPT-4.0, to generate sentence-level representations for the identified topics. These sentence-level representations provide richer context and more interpretable descriptions, enabling a deeper understanding of the topics.

Textbox 2. **The prompt structure and 1 example to find topics description.**

Topic description prompt structure:

 System role:

**  ** Topic main words: [Top 10 words]

**  **Topic document examples: [5 closest examples to the center of the topic]

 User role:

   “Describe topic in a short phrase?”

Topic description prompt example:

System role:

   Topic main words: [“masks,” “mask,” “face,” “wearing,” “wear,” “use,” “oxygen,” “hypoxia,” “cause,” and “you’'].

Topic document examples:
  1. Centers for Disease Control and Prevention (CDC) does not recommend wearing masks.
  2. The US CDC contradicted itself by advising people to wear cloth masks against the novel coronavirus while also saying masks do not stop smoke inhalation during a wildfire.
  3. The World Health Organization changed its mind about masks and now says that they can increase the risk of infection.
  4. Nonmedical masks are ineffective in preventing the spread of the disease, are circulating online.
  5. Whether CDC had scheduled announcement that all should wear masks for everyday life.

User role: “Describe topic in a short phrase?”

Output answer: “Controversies and debates over mask wearing and its effectiveness”

The prompt includes the top 10 most representative words for each topic as identified by the topic modeling algorithm, and to add context and depth to the topic descriptions, we select 5 documents that are closest to the center of the corresponding topic cluster. The selection of these documents is guided by cosine similarity, performed using equations 1 and 2, which measure the proximity of documents to the cluster center in the semantic space. An example of this process is provided in Textbox 2, illustrating how the top words and representative documents are integrated into the prompt to produce a high-quality sentence-level representation.

By combining these elements, we construct detailed and context-rich prompts that guide ChatGPT-4.0 in generating coherent and semantically accurate sentence-level topic representations. This approach ensures that the abstract themes identified by topic modeling are translated into human-readable and interpretable descriptions.

To evaluate the quality of the generated topic descriptions, we engaged 3 independent raters to assess the descriptions based on 3 categories: appropriate, somewhat appropriate, and not appropriate. The evaluation results are shown in Table 4 and highlight that the majority of topic descriptions were well received. Specifically, the total proportion of accepted descriptions (the sum of those rated as appropriate and somewhat appropriate) was 99.6%. This acceptance rate demonstrates the effectiveness and reliability of the proposed method for generating meaningful and contextually relevant topic descriptions. There was perfect agreement in 144 out of 169 (85.2%) of the sentences. Two out of 3 raters agreed on category in 24 out of 169 (14.2%) of the sentences. There was a single instance in which no raters agreed in 1 out of 169 (0.6%) of the sentences.

**Table 4.** Percentage of topic descriptions rated as “appropriate,” “somewhat appropriate,” and “not appropriate” by each rater, along with the total number of accepted topic descriptions, calculated as the sum of those rated “appropriate” and “somewhat appropriate.”

Raters	Appropriate (%)	Somewhat appropriate (%)	Not appropriate (%)	Total accepted (%)
Rater 1	98.23	1.77	0	100
Rater 2	94.67	5.33	0	100
Rater 3	89.94	8.88	1.18	98.82
Average	94.28	5.32	0.39	99.6

After generating concise descriptions for each topic, we used the structured prompt framework outlined in Textbox 3, which includes a list of these topic descriptions. This structured prompt was then input into the ChatGPT-4.0 API to further refine and categorize the topics into overarching themes.

Textbox 3. **Structure of the prompt for identifying topic themes.**

Finding topic themes prompt structure:

 System role:

  The following are topics related to COVID-19 pandemic. Go through all topics and categorize them into relevant groups. Mention topics number for each category.

 User role:

  Topics description list

The output from this process not only provides a clear categorization of topics into distinct themes but also includes a concise description for each theme. This step ensures that the topics are grouped in a meaningful and interpretable way, facilitating a deeper understanding of the data’s thematic structure.

**Figure 4.**
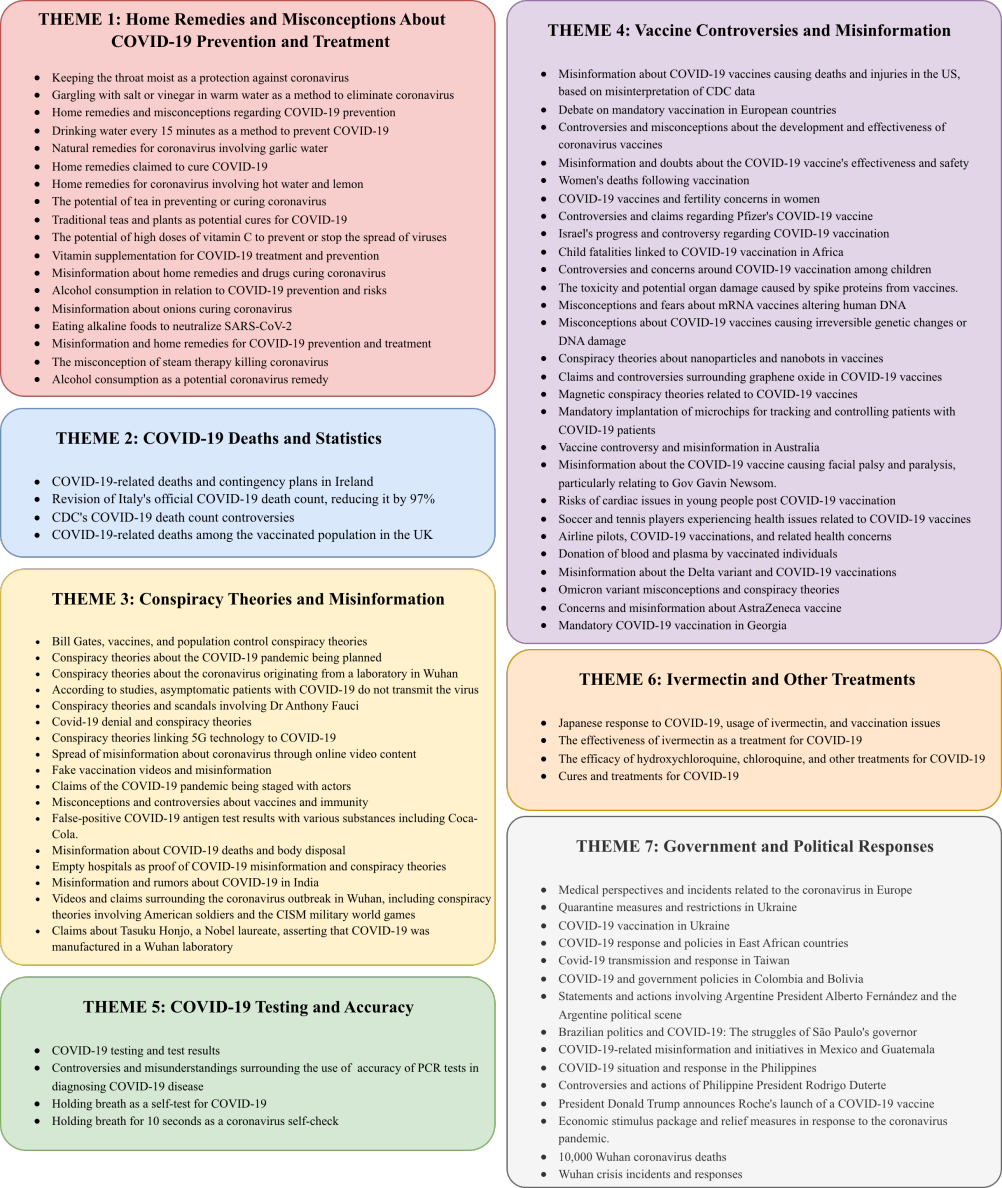
Descriptions of themes 1-7 along with the corresponding topic descriptions assigned to each theme. CDC: Centers for Disease Control and Prevention.

The categorized topics and their corresponding theme descriptions are provided in Figures4  5, showcasing the effectiveness of the proposed method in generating coherent and insightful thematic groupings.

**Figure 5.**
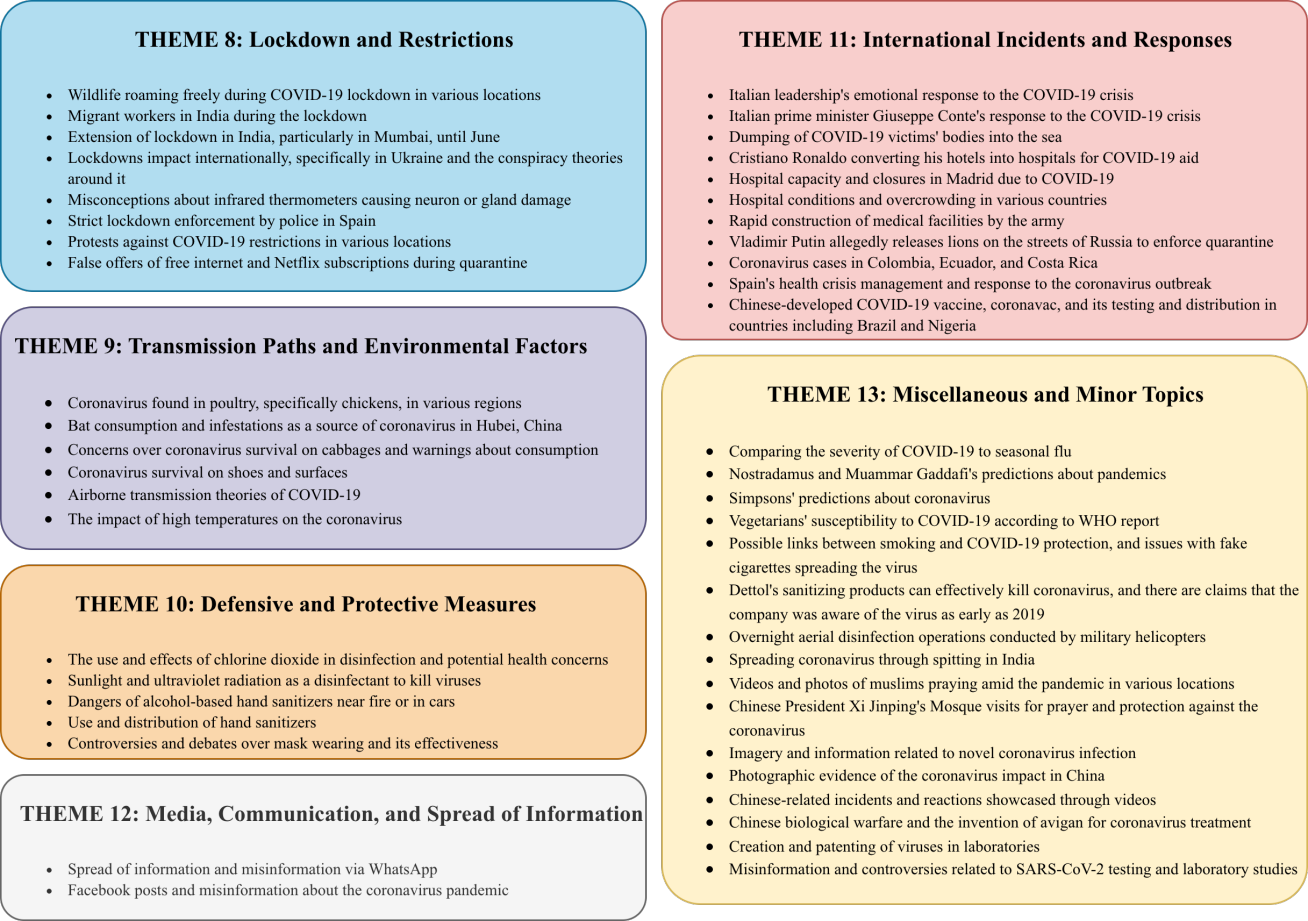
Descriptions of themes 8-13 along with the corresponding topic descriptions assigned to each theme.

Using the algorithm in Textbox 1, we assign each document to a topic, enabling us to determine the distribution of each theme. Table 5 shows the distribution of all themes, with theme 4 (Vaccines) and theme 3 (Conspiracy Theories) emerging as the first and second most prevalent misinformation themes. Here are all the themes and their percentages:

**Table 5.** Distribution of COVID-19 misinformation themes.

Theme	Values (N=18,018), n (%)
Theme 1: Home remedies	1334 (7.40)
Theme 2: Deaths and statistics	570 (3.16)
Theme 3: Conspiracy theories	3459 (19.20)
Theme 4: Vaccine	3595 (19.95)
Theme 5: Testing	546 (3.03)
Theme 6: Ivermectin	814 (4.52)
Theme 7: Government	2094 (11.62)
Theme 8: Lockdowns	1316 (7.30)
Theme 9: Transmission	297 (1.65)
Theme 10: Defensive	1269 (7.04)
Theme 11: International	891 (4.95)
Theme 12: Media	252 (1.40)
Theme 13: Minor topics	1581 (8.77)

**Figure 6.**
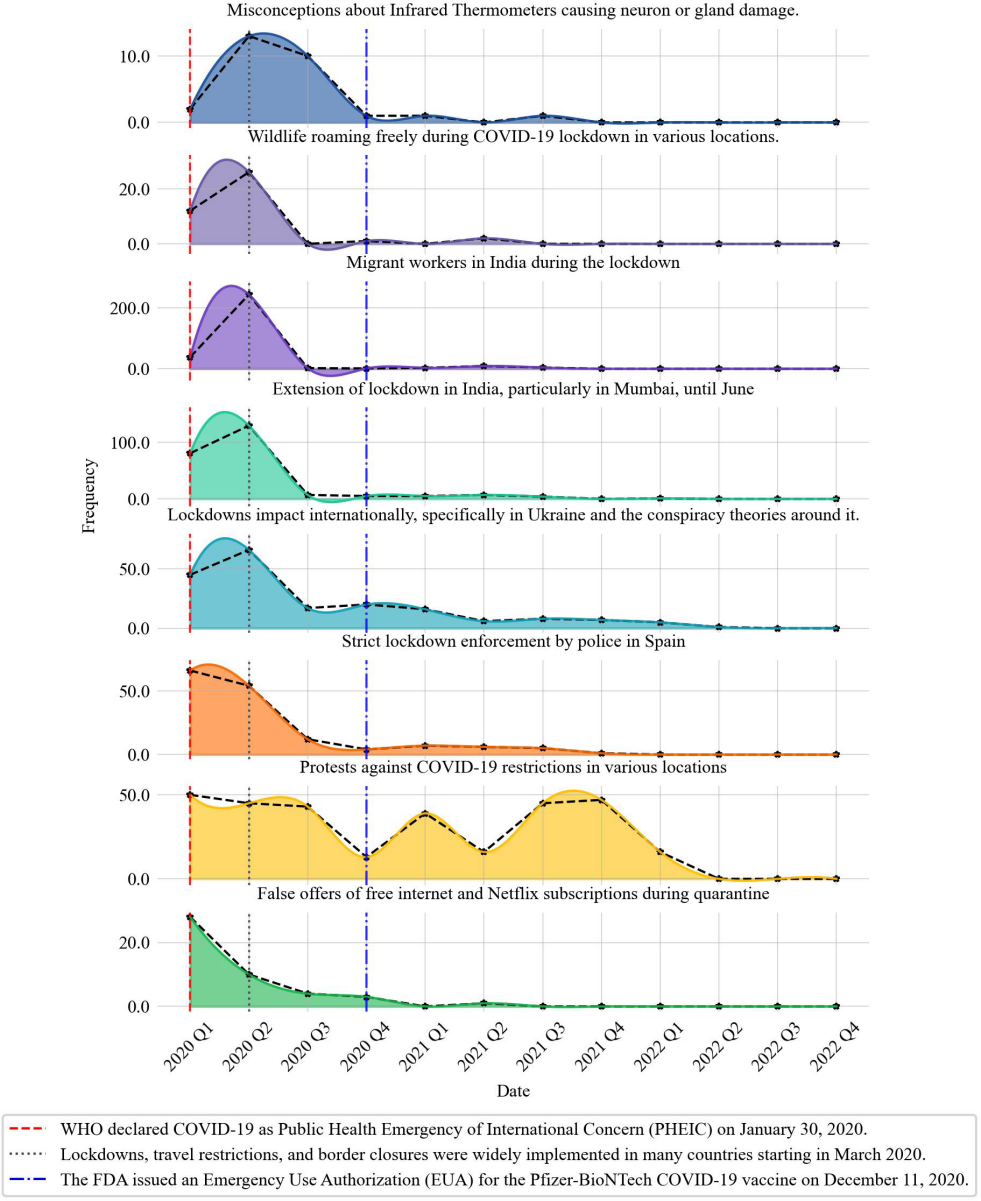
Distribution of theme 8 topics during the time. FDA: Food and Drug Administration; WHO: World Health Organization.

Figure 6 shows the distribution of each topic within theme 8 over time, illustrating that protests against COVID-19 restrictions were the only misinformation topic that actively persisted even after the release of the COVID-19 vaccine.

After identifying the misinformation themes, we leverage the explanations provided in the IFCN dataset as a basis to draw refutation arguments to address these themes. For each identified theme, we develop a refutation that aligns with its context, aiming to clarify the nature of the misinformation, its potential origins, and its impact. These refutations are shown in Textbox 4. The refutation arguments list for 13 themes, providing valuable insights into the underlying reasons for the misinformation.

Textbox 4. **The refutation arguments list for 13 themes.**

Refutation list:

1. This content may contain misinformation related to home remedies for COVID-19 prevention and treatment.
2. This content may contain misinformation related to COVID-19 deaths, statistics, and their relation to vaccination.
3. This content may contain conspiracy theories and misinformation related to COVID-19, including unverified claims, distorted facts, or manipulated content.
4. This content may contain misinformation related to COVID-19 vaccines, including false claims about safety, efficacy, and side effects.
5. This content may contain misinformation related to COVID-19 testing accuracy, including false claims about polymerase chain reaction tests and unscientific self-check methods.
6. This content may contain misinformation related to COVID-19 treatments, including exaggerated claims about ivermectin, hydroxychloroquine, or unproven supplements.
7. This content may contain misinformation related to government and political responses to COVID-19 pandemic, including distorted facts, fabricated claims, or misrepresentation of policies and actions.
8. This content may contain misinformation related to COVID-19 lockdowns and restrictions, including fabricated or misrepresented events, videos, or claims.
9. This content may contain misinformation related to COVID-19 transmission and survival in various environments, including unverified claims about foods, surfaces, or environmental factors.
10. This content may contain misinformation related to defensive and protective measures against COVID-19 pandemic, including false or exaggerated claims about masks, sanitizers, UV rays, or disinfectants.
11. This content may contain misinformation related to international incidents and responses to COVID-19 pandemic, including fabricated reports of government actions, health care capacity, or global cooperation.
12. This content may contain misinformation related to the spread of COVID-19 information on social media and messaging apps, including false claims about government policies, platforms, or media manipulation.
13. This content may contain miscellaneous misinformation related to COVID-19 pandemic, including distortions about products, religious practices, bioweapons, and other fabricated claims.

### Provide Refutation

In the final stage of our process, we design a prompt text to enable ChatGPT-4.0 to detect specific misinformation themes. The prompt text includes a detailed description of the themes and a question-answer list. To create this question-answer list, we select the document closest to the center of each topic and associate it with the corresponding theme. Detailed information about the prompt text can be found in Textbox 5.

Textbox 5. **Prompt structure and 1 example to find a document theme.**

Finding document themes prompt structure:

 System role:

  “The following is the description of topic themes related to COVID-19 misinformation. Find the closest theme for the given text. Answer in a consistent style.”

 User role:

  Themes description list

 Assistant role:

  Question-answer list

 User role:

  Input text

Finding topic themes prompt example:

 System role:

  “The following is the description of topic themes related to COVID-19 misinformation. Find the closest theme for the given text. Answer in a consistent style.”

 User role:

  Themes description list

 Assistant role:

  Question-answer list

 User role:

  “A video shows that Bill Gates admits the vaccine will no doubt kill 700,000 people.”

 Output answer:

  Theme 3: “Conspiracy Theories and Misinformation”

**Figure 7.**
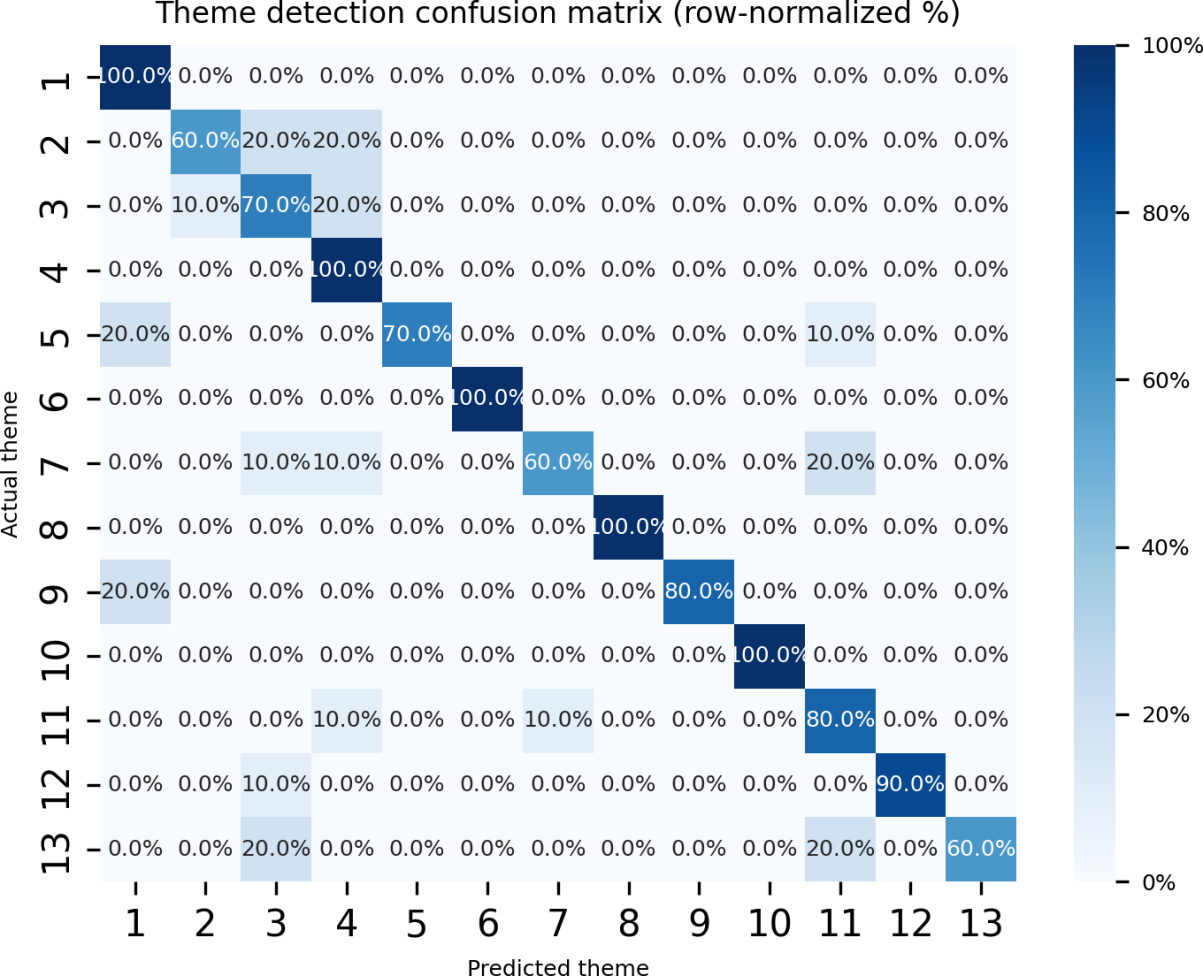
Confusion matrix of theme detector.

To evaluate our approach, we randomly selected 130 (10 documents per theme) documents from the IFCN dataset that are not included in the question-answer list. We then tested the prompting method with ChatGPT-4.0, achieving an 82% accuracy rate in detecting the correct themes. Moreover, as showing in Figure 7 the model struggled to detect themes 2, 7, and 13 in comparisons with other themes.

### Proposed System

Based on the process we introduced, we propose the development of an MDIS. This system is designed to first determine whether a given text document contains misinformation or not. If the document is identified as containing misinformation, the system then detects its theme and provides a detailed refutation of the misinformation. The primary objectives of MDIS are to prevent the spread of misinformation and to enhance public health knowledge.

MDIS operates by integrating 3 key components. First, it uses a trained LLM to classify text documents as either misinformation or nonmisinformation. Next, it uses another trained LLM to detect the specific misinformation theme within documents identified as containing misinformation. Finally, it leverages a refutation list, which is generated during the theme description phase, to provide context and counternarratives for each detected theme. This comprehensive approach enables the system to effectively address misinformation while equipping users with accurate information.

Figure 1C illustrates an example of an input to MDIS and its corresponding output, demonstrating how the system analyzes a document, identifies misinformation, detects the associated theme, and presents an explanatory response.

## Discussion

### Principal Results

This study developed the MDIP, a structured workflow for analyzing health misinformation and generating explanatory counterarguments. Building on MDIP, we designed a prototype MDIS that combines detection, topic detection, theme identification, and refutation generation. While the system has not yet been deployed in real-world environments, the results illustrate its potential to support public health communication.

In the misinformation classification task, LLMs achieved reasonable performance, with BERT reaching 98% accuracy. Enriching training datasets also reduced false positives on AI-generated misinformation by 44% compared with prior baselines, suggesting improved robustness across different linguistic styles. Topic modeling experiments highlighted the advantages of BERTopic relative to LDA and Top2Vec, with higher coherence and diversity metrics. To address the issue of unassigned documents, the algorithm in Textbox 1 was used to assign outliers to their nearest topic cluster, thereby improving coverage of the dataset. To enhance interpretability, word-level topic outputs were converted into sentence-level descriptions through prompt engineering with ChatGPT-4.0. These descriptions were judged appropriate or somewhat appropriate in 99.6% of cases by independent raters, indicating the value of sentence-level representations for clarity and interpretability. Building on these descriptions, topics were further grouped into broader themes and linked with theme-specific refutations.

Finally, a prompt-based detector for misinformation themes achieved 82% accuracy. These results demonstrate how detection, topic modeling, thematic grouping, and refutation can be integrated into a single workflow. The prototype system (MDIS) represents an illustration of concept, and while the findings are encouraging, validation is limited to English language, COVID-19–related data, and offline testing. Broader generalization, multilingual adaptation, integration into health communication workflows, and longitudinal evaluation remain important directions for future work.

### Limitations

While this study presents a framework for detecting and addressing misinformation, several limitations should be acknowledged. First, the system’s performance relies on the quality and diversity of the datasets that were used for training and topic modeling, which may not fully capture the linguistic and contextual nuances of misinformation in different regions, languages, or cultural contexts. This limitation introduces a potential bias: refutations that appear clear and persuasive in one sociocultural context may be ineffective—or even counterproductive—in another.

The model has been tested solely on English text–based misinformation, and it has not been evaluated for multilingual and multimodal adaptation, meaning its ability to detect misinformation and provide persuasive refutation across different languages and sociocultural contexts remains uncertain. Moreover, while the theme detection module achieved an accuracy of 82%, this leaves an 18% error margin, especially in ambiguous or overlapping themes, which can lead to inaccuracies in refutation and reduce the overall effectiveness of the generated warning texts. Developing and integrating more sophisticated algorithms to address overlapping in topic themes could substantially enhance the accuracy of theme detection. Improved theme separation would not only yield clearer and more coherent thematic structures but also strengthen the generation of precise and contextually relevant refutations. In turn, this refinement would enable more effective countermeasures against health misinformation, thereby improving the system’s overall capacity to support public health communication and trust. False positives and negatives remain a concern, particularly when misinformation contains opinion-based, satirical, or context-dependent elements. Although the generated refutations follow a systematic structure, they may not always be contextually relevant, persuasive, or ethically suitable for diverse audiences. In the absence of a human-in-the-loop or oversight mechanism, the system may produce counterarguments that fail to resonate with users or could be perceived as unreliable. In addition, the system has not yet been extensively tested in real-world applications, which limits understanding of its practical impact on misinformation spread and public health outcomes. Furthermore, misinformation evolves over time, and a model trained on past narratives may require periodic retraining to remain effective against emerging falsehoods, including AI-generated misinformation. Using pretrained LLMs such as ChatGPT depends on the current version of the model and its accessibility to users. Therefore, it is necessary to update the system regularly when the model is changed or becomes unavailable. Moreover, since passing datasets through third-party platforms may compromise the security of the framework, future work could focus on developing an in-house solution by training a dedicated model for our specific tasks, thereby eliminating the reliance on external platforms. Finally, the reliance on automated methods raises potential concerns about interpretability and transparency, which are crucial for fostering trust and adoption by end users.

### Comparison With Prior Work

The proposed MDIP and the resulting MDIS build upon and advance the body of research focused on misinformation detection and mitigation. The proposed method transforms raw posts into *actionable units*—sentence-level topic labels, aggregated themes, and paired refutations—linking detection outputs directly to message design and response playbooks used by health teams. Previous research has demonstrated the efficacy of ML models, particularly deep learning approaches, in detecting misinformation. They used ML techniques to classify fake news using textual features, demonstrating the value of automated detection methods [49,52,69]. Our study extends these efforts by integrating enriched datasets containing both formal and informal language styles, ensuring better generalization across diverse linguistic sources, including AI-generated misinformation.

Topic modeling techniques such as LDA have been used in prior studies to analyze misinformation [35,53,55]. Our approach improves on these works by addressing limitations in document assignment and theme interpretation. We used an algorithm to assign every document to the most relevant topic, resolving the common issue of unclassified documents in topic modeling. In addition, we moved beyond word-level topic representations to generate sentence-level descriptions, offering richer and more interpretable insights. By tracking shifts in sentence-level topics and theme distributions, communicators can conduct pre-/postassessments of campaigns or platform policy changes, complementing survey-based outcomes. Finally, we designed an effective prompt text to automatically identify the themes of misinformation. This automated approach reduces reliance on manual interpretation, minimizing human bias and increasing scalability.

Many prior studies have addressed misinformation detection or topic analysis in isolation. They analyzed misinformation using sentiment analysis but did not integrate detection with thematic analysis and did not provide a framework for counteracting misinformation [11,35,70]. Our work unifies detection, topic modeling, thematic refutation, and public health intervention in a single framework. The MDIS framework automates the end-to-end process, offering a scalable solution to tackle the complexity of misinformation dynamics.

### Conclusions

This work contributes a methodological framework for infodemiology and digital health operations. We transform misinformation into actionable units—themes and refutations—so that health teams can act (communicate, triage, and evaluate). Moreover, analyzing misinformation using a hierarchical (2-level) sentence-level description and assigning all documents to topics makes it possible to observe theme and topic distributions over time, providing a broad and sensible overview of misinformation. Sentence-level topics and theme distributions serve as measurable indicators for surveillance and intervention evaluation (eg, pre-/postcampaign shifts and surge detection). We introduce MDIP and MDIS that enable rapid response playbooks and reduce analyst workload. To support adoption, we release prompt templates and code as implementation artifacts that teams can readily adapt. Real-world deployment, however, requires governance mechanisms (human-in-the-loop review and audit logs), multilingual extensions, and prospective trials with health agencies or platforms to quantify downstream impact (eg, reduced spread and improved literacy). Ultimately, these contributions orient detection toward operational use—prioritizing interpretability and intervention design—so that public health actors can move from finding misinformation to effectively countering it.

## Abbreviations

AI: artificial intelligence
BERT: Bidirectional Encoder Representations from Transformers
CDC: Centers for Disease Control and Prevention
CV: Coherence Value
GPT-2: Generative Pre-trained Transformer 2
HPV: human papillomavirus
IRBO: Inverted Rank-Biased Overlap
LDA: Latent Dirichlet Allocation
LLM: large language model
MDIP: Misinformation Detection and Inoculation Process
MDIS: Misinformation Detection and Inoculation System
ML: machine learning
NPMI: Normalized Pointwise Mutual Information
T5-base: Text-to-Text Transfer Transformer
WHO: World Health Organization
